# Correction: Factors associated with SARS-COV-2 positive test in Lifelines

**DOI:** 10.1371/journal.pone.0302619

**Published:** 2024-04-19

**Authors:** Grigory Sidorenkov, Judith M. Vonk, Marco Grzegorczyk, Francisco O. Cortés-Ibañez, Geertruida H. de Bock

In Fig 1, the number of participants in the control group and the number of balanced datasets are incorrect. Please see the correct Fig 1 here.

**Fig 1:**
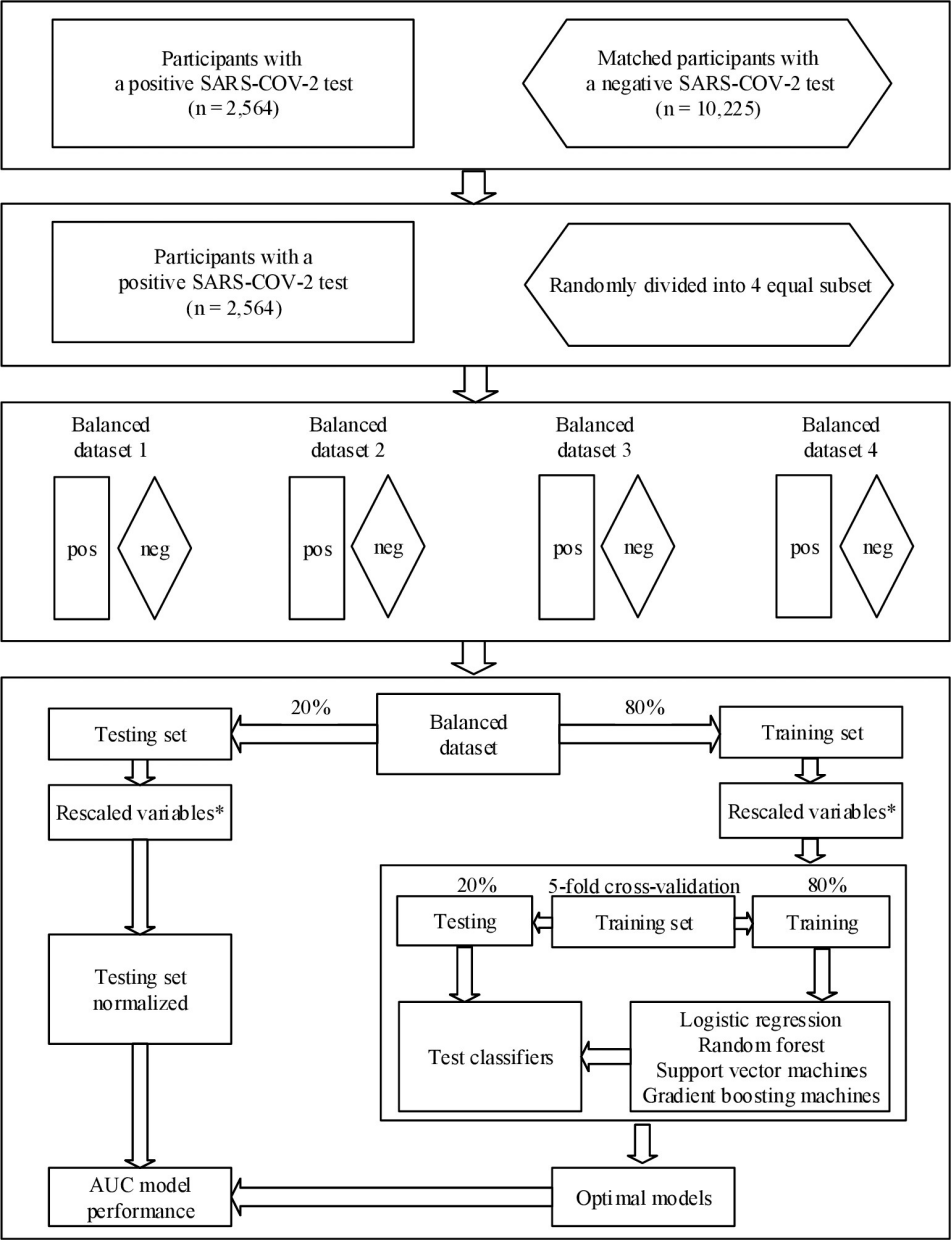
Overview of the procedure followed to reduce class imbalance (equalization strategy). * Continuous variables were rescaled to the interval between zero and one; AUC–Area Under the Curve.
