# Supplementary material for: Factors associated with SARS-COV-2 positive test in Lifelines
Source: PLoS One. 2023 Nov 29;18(11):e0294556. doi: 10.1371/journal.pone.0294556 (PMC10686451; doi:10.1371/journal.pone.0294556)
Supplement: Attachment — Submitted filename: Point-by-point_reply.docx [file pone.0294556.s002.docx]

Dear Editor,

Thank you for the opportunity to improve our manuscript. We appreciate the given feedback and good suggestions. We have prepared the responses to the comments point by point, which you can find below. We submitted two versions of the manuscript. One was with revisions marked using tracked changes and another was a clean manuscript after revision. The revisions have been drafted in consultation with all coauthors, and each author has given approval to the final form of the revisions.

Thank you for your consideration.

On behalf of authors,

Kind regards,

Grigory Sidorenkov

University Medical Center Groningen, Department of Epidemiology

Groningen, the Netherlands, PO Box 30.001, FA 40, 9700 RB.

E-mail: g.sidorenkov@umcg.nl.

Tel: +31 50 361 0739 (Secretariat).

Reviewer #1:

Comment 1. Abstract: 2564(12%) or 21964 is not of ?

*Authors’ reply: Thank you for pointing out. We have corrected the sentence.*

Comment 2. Perspective or Limitation needs to be commented on the association with genetic vaccination. Differences by vaccination or not, by number of vaccinations, and the point that PCR positivity is higher within 14 days after vaccination due to immunosuppression.

*Authors’ reply: Following the advice of the reviewer, we have addressed this point in the limitations and added the following text on page 9: “The probability of a positive result of a PCR test for SARS-COV-2 is expected to be higher within 14 days after vaccination due to immunosuppression. Patients who experience symptoms within 14 days before a positive PCR test can be expected to have lower vaccination rates, therefore, the association between the presence of symptoms and a positive SARS-COV-2 test can be underestimated. However, in our study we only used the data until April 2021. At that time only a minority of the population was vaccinated.”*

Comment 3. Since the Omicron strain, viral infection has been localized (nose and throat) and sore throat has become more pronounced.

*Authors’ reply: We have added at the limitation section on page 10 that “this study looks at the period when the ‘alpha’ variant of SARS-COV-2 was prevalent in the Netherlands and self-tests were not available at that time, making the findings less relevant for the currently prevalent variant of the virus”, which is Omicron.*

Comment 4. The report only that smoking is less likely to be PCR positive misleads the reader.

It should be added that a history of smoking is associated with more severe COVID19.

*Authors’ reply: We agree with the reviewer that this is an important addition. We have added the following text in the discussion section on page 10: “However, it is important to mention that a history of smoking is associated with more severe COVID19 [28].”*

Reviewer #2: This paper studies factors associated with SARS-COV2 positive tests in a cohort from The Netherlands, which was examined using questionnaires. Below are major and minor comments to the paper.

Major comments

Comment 1: The overall aim of the paper appears unclear to me. In the title of the paper, focus is on "factors associated with...", whereas the paper speaks about risk factors for a positive test (l1, p4). Authors should clarify if it is merely associations or risk (causal?) they are concerned with, and if "risk factors" is the choice, it should be clarified how a symptom of Covid-19 can be a risk factor (for a positive test)?

*Authors’ reply: We acknowledge that the term 'risk factors' may not be suitable for our study and have revised the manuscript accordingly. We are referring to factors that may not necessarily have a causal relationship with a positive SARS-CoV-2 test. We also made a remark about it in the introduction on page 4 adding the following sentence: “It should be noted that the investigated factors may not have a causal relationship with a positive SARS-COV-2 PCR test but could lead to a better defined indication for SARS-COV-2 PCR-testing and, as a result, reduce the number of people requiring a test”.*

Comment 2: The previous item also has implications for the intended use of the results, which I find unclear. It seems that authors suggest their finding could be used to create some sort of high risk screening strategy instead of the broad population-based screening used in many countries in an effort to combat Covid-19. If such a high risk screening strategy is indeed the objective, authors should have focused much more on estimating the usual quality measures of a screening test such as sensitivity, specificity, positive and negative predictive values. Further I am missing considerations of the fact that since Covid-19 is a contagious disease, sensitivity or capture rates are probably much more important than specificity? If the intention is not to suggest a high-risk screening strategy, the scientific value of the paper is likely less and in any case needs to be elaborated.

*Authors’ reply: We appreciate the reviewer for giving us this valuable advice. We have reported sensitivity and specificity and have given more attention to it in the text.*

*In the introduction on page 4 we added the following text: “Moreover, the secondary aim of this study was to determine the sensitivity and specificity of the model including the identified factors in terms of predicting a positive SARS-COV-2 PCR test.“*

*In the methods section on page 6 we added the following text: “The overall performance of the models reported as AUC and its confidence interval (CIs). Sensitivity and specificity reported to provide information about the model's ability to correctly identify positive and negative cases and discriminate between them.”*

*In the results section on page 7 we added the following text: “With a sensitivity of 81% the model can identify people with a positive SARS-COV-2 test and with a specificity of 90% the model can identify the participants with a negative SARS-COV-2 test. The overall performance of the logistic regression models was good (AUC = 0.86).”*

*In the discussion section on page 8 we added the following text: “The model including the identified factors can identify the participants with a positive SARS-COV-2 test with a moderate sensitivity of 81% and the participants with a negative SARS-COV-2 test with a relatively high specificity of 90%.”*

Comment 3: The fundamental data source is created by participants filling out a questionnaire. This raises profound questions about the validity of the self-reported data. This is only mentioned very briefly in Limitations. I fail to understand why the prevalence of a positive test is likely over-estimated with these data? I also fail to see why this in itself is a problem, if focus is on the associations with other self-reported (risk) factors? In other words, I think authors should discuss the potential for bias in these associations due to the self-reporting. Also concerning self-reported values: The first sentence of the Results state that "...21,964 patients were tested...", but would it be more precise to say that this number said they had been tested? Or is there some external data which supports this number?

*Authors’ reply: We appreciate the reviewer's comment on the validity of self-reported questionnaires and acknowledge that it is difficult to determine if test positivity is reported differently than other data. In our study, we assumed that due to the media coverage and heightened attention to the topic, some individuals may have overreported a positive SARS-COV-2 PCR test. However, it has not been supported by evidence, therefore, we omit such a reporting. In the limitation we have mentioned the following: “It is important to note that the data were self-reported, which can lead to overreporting of symptoms related to SARS-COV-2 due to increased media attention, such as loss of sense of smell or taste and cough [29]”. Furthermore, we have rephrased the first sentence in the results following the remark of the reviewer: “In total, 21,964 of 71,992 participants who completed at least one COVID-19 questionnaire reported that they were tested between June 2020 and April 2021, of which 2,564 (12%) had a positive SARS-COV-2 test”.*

Comment 4: I think authors need to discuss potential bias from self-selection by invited participants - how many chose to participate and did they differ from those who did not participate? How may this have affected the results?

*Authors’ reply: We have added the following sentence to the limitation section on page 9: “Though there was a small significant difference between the non-responders (29%) and responders (71%) in age, sex, BMI, and smoking status [12], it is not expected to impact the findings of this study.”*

Comment 5: I am not sure I understand the study design and why it has been used. First, the first positive PCR test for each participant is selected, if any, and then the first negative test for all other participants? This must mean that "controls" are selected conditional on not later becoming "cases", but this seems problematic from an epidemiological point of view as it conditions on the future. Perhaps it would be more straightforward to use a standard case-control study design, possibly with matching? This could also address the large variation in testing strategies over time.

*Authors’ reply: We acknowledge that the selection of controls conditional on their future status may be seen as a limitation of this study. However, we chose this approach to address the research question using longitudinal data. We did not select controls but consider all non-cases in a study period to be controls. We do not expect it would bias the results of this study, because the data about the SARS-COV-2 test and other factors were in most cases collected from the questionnaire at the same time point. It was common, that people received multiple tests for SARS-COV-2 infection during the study period. Using a case-control design with matching may be another way of dealing with the data, however, it could also introduce other biases, such as selection bias.*

Comment 6: The analytic strategy is confusing in my view. The main analysis is logistic regression with automated selection of important explanatory factors. This is then compared to three other automated algorithms used in machine learning. However the first results presented (Figure 1) are estimates from logistic regression of the effect of individual factors with confidence intervals. Since an automated selection process has been used, we know a priori that only larger effects have been included and that confidence intervals are downward biased. If focus is on valid estimation of effect sizes this should be based on a pre-specified analysis plan without data-based selection of which effects to consider.

*Authors’ reply: We acknowledge that CIs may be slightly biased downwards due to the automatic selection of the variables, but we do not expect such bias to be relevant, since only a few variables were excluded by the automatic selection. We consider selection based on AIC as an optimal way of selecting relevant variables in our analysis. To ensure that our logistic regression model performed well, we compared its performance to three different nonlinear algorithms, which was comparable.*

* Minor comments

Comment 7: p6, top: It has not yet been defined what these subsets refer to.

*Authors’ reply: We thank the reviewer for pointing this out. We have shifted the sentences about the missing data and the way of dealing with it to the end of the ‘analysis’ subsection to satisfy the logical order with regard to the subsets definition.*

Comment 8: p6, top: How many imputed datasets were created?

*Authors’ reply: On page 6 we now clarified that “A total of five imputed datasets were generated per subset, which were then pooled for the analysis.”*

Comment 9: p6, middle: Why should there be a balanced number of participants with a positive and negative PCR? Logistic regression estimates are unbiased by this imbalance in ordinary statistical analyses, so please explain why this should be different when labelled machine learning?

*Authors’ reply: Class imbalance can lead to bias in machine learning because it can cause the model to favor the majority class and ignore the minority class. This results in poor performance of the model in predicting the minority class, which is the number of participants with a positive SARS-COV-2 test. In the case of class imbalance, sensitivity is more affected than specificity. This is because the minority class include fewer participants, and the model tends to have a higher false negative rate, resulting in lower sensitivity. On the other hand, specificity remains high as the majority class has more participants. Class imbalance can cause the AUC to be artificially high, which can give a false sense of model performance. Therefore, it is important to consider both sensitivity and specificity in addition to AUC when evaluating the performance of a model in the presence of class imbalance. As the number of participants with a negative SARS-COV-2 test is 8 times larger, we randomly selected 8 equal subsets from those. We have now added the reference [14] to a paper on page 6 that describes the bias potential of class imbalance in machine learning.*

Comment 10: p9, top: The conclusion is overstated in my view as it gives the impression is warranted for patients with some characteristics, thereby implying implicitly that it is not warranted for others. If the objective of testing is containment of the number of infected, we need knowledge about how few it would be okay to miss with a testing strategy, before we can reach a conclusion that restricting testing to certain groups with higher risks of testing positive is appropriate. I realize that such knowledge may be largely missing, but it is nevertheless a prerequisite here.

*Authors’ reply: We have rephrased the conclusion to avoid too strong wording: “In conclusion, people who have been in contact with another positive person or those with loss of smell and taste have a much higher risk of being tested positive for SARS-COV-2 infection.”*
