# Supplementary material for: Factors associated with SARS-COV-2 positive test in Lifelines
Source: PLoS One. 2023 Nov 29;18(11):e0294556. doi: 10.1371/journal.pone.0294556 (PMC10686451; doi:10.1371/journal.pone.0294556)
Supplement: Attachment — Submitted filename: Point-by-point_reply.docx [file pone.0294556.s003.docx]

Dear Editor,

Thank you for the opportunity to improve our manuscript. We appreciate the given feedback and good suggestions. We have prepared the responses to the comments point by point, which you can find below. We submitted two versions of the manuscript. One was with revisions marked using tracked changes and another was a clean manuscript after revision. The revisions have been drafted in consultation with all coauthors, and each author has given approval to the final form of the revisions.

Thank you for your consideration.

On behalf of authors,

Kind regards,

Grigory Sidorenkov

University Medical Center Groningen, Department of Epidemiology

Groningen, the Netherlands, PO Box 30.001, FA 40, 9700 RB.

E-mail: g.sidorenkov@umcg.nl.

Tel: +31 50 361 0739 (Secretariat).

Review Comments to the Author

Reviewer #2: Following the revision, the paper is now much easier to follow and in general authors have addressed the points I have raised. There is however one issue remaining, which I find crucial as it pertains to the study design. I appreciate that authors now have made the study design clear, but I think it also highlights how it is unusual and will by necessity have to influence the interpretation and conclusions of the study. The issue is with how controls have been selected.

Authors have selected controls as those never testing positive within the follow-up. I raised the concern that it might be problematic since it conditions on the future - I still think that is valid criticism, and I find authors' statement unconvincing, when they just note that this is what they chose to do (response letter, reviewer II, reply to comment 5). With the clearer description of the study design, I would like to strengthen my critique here: The participants compared are those testing positive (at least once) with those never testing positive (within the study follow-up). If authors want to keep this, I think the specificity no longer concerns an individual with true negative disease status at the time of a single test, but it is the specificity of individuals who are truly negative at the time of all PCR tests within the study. I think this is a less relevant question when considering the aim of finding factors associated with single test results. I would therefore much prefer that authors used a (matched) case-control study design to select relevant negative tests without conditioning on the future of individual patients test history.

*We acknowledge that the selection of controls conditional on their future status may be seen as a limitation of this study. We revised the paper where necessary and used case-control study design as suggested by the reviewer. Cases comprised individuals who tested positive for SARS-COV-2, while controls were individuals who tested negative for the infection. The first positive test within the study period was selected as the case for each participant. Controls were then matched to cases based on the timing of their SARS-COV-2 tests, maintaining a 1:4 ratio. This is also reported in the methods sectionof the paper on pages 4 and 5.*

*The changes only slightly affected the findings. Of the specific changes, we would like to point out the changes in the magnitude of the sensitivity and specificity values, where sensitivity is increased ot relatively high (88.4) and sensitivity decreased to moderate (81.1).*

*All the adjustments were highlighted in the text.*
